# Novel Duck Orthoreovirus σA Protein Inhibits Interferon Signaling by Impeding STAT1/STAT2 Nuclear Translocation

**DOI:** 10.1155/tbed/8440800

**Published:** 2025-09-15

**Authors:** Boyi Xu, Chenchen Jiang, Lei Di, Lan Zhou, Zhouyuan Wang, Yi Tang, Rendong Fang, Hongzhi Wang

**Affiliations:** ^1^College of Veterinary Medicine, Southwest University, Beibei District, Chongqing, China; ^2^Chongqing Customs Port Outpatient, Chongqing International Travel Healthcare Center, Yubei District, Chongqing, China; ^3^Kunming Hemeihua Feed Limited Company, Kunming, Yunnan Province, China; ^4^Institute of Animal Sciences, Chinese Academy of Agricultural Sciences, Haidian District, Beijing, China

**Keywords:** σA protein, interferon, JAK-STAT signaling pathway, novel duck orthoreovirus, nuclear translocation

## Abstract

Novel duck orthoreovirus (NDRV) infection induces severe splenic necrosis and immunosuppression in ducks, leading to substantial economic losses in the duck farming industry. While the avian orthoreovirus (ARV) σA protein is known to exhibit interferon (IFN) antagonistic activity, whether NDRV possesses a similar function and the underlying molecular mechanisms remain unclear. This study demonstrates that NDRV not only counteracts the antiviral clearance effect of type I IFN (IFN-I) but also markedly suppresses IFN-mediated signal transduction. Further investigations revealed that the NDRV σA protein specifically inhibits IFN signaling and its associated antiviral effects by blocking the nuclear translocation of STAT1/STAT2, thereby, facilitating innate immune escape. This discovery elucidates for the first time a novel mechanism by which the NDRV σA protein regulates the JAK-STAT signaling pathway. These findings provide a theoretical foundation for the development of antiviral strategies targeting immune regulation.

## 1. Introduction

Duck orthoreovirus (DRV)-induced splenic necrosis in ducks has been prevalent in duck farms across China since 2006 [1]. Clinical observations indicate that, although the direct mortality rate of DRV infection is relatively low, the actual mortality can reach 10%–15% due to immunosuppression induced by DRV, which frequently leads to secondary infections [2, 3]. With the ongoing evolution of viruses, variants exhibiting significantly enhanced pathogenicity have emerged in recent years [4]. In 2017, our research group was the first to report the emergence of a novel DRV (NDRV) [5]. The NDRV strains cause characteristic severe splenic necrotic lesions, increased pathogenicity, and higher clinical mortality rates [6]. NDRV infection compromises the host immune system through its rapid transmission and potent pathogenicity, resulting in dual detrimental effects it not only increases the risk of secondary infections but also interferes with the efficacy of conventional vaccines, thereby, causing substantial economic losses to China's duck farming industry [7, 8].

Avian orthoreovirus (ARV) is an important member of the *Orthoreovirus* genus with a genome consisting of 10 segmented double-stranded RNA (dsRNA) segments [9]. The S1 gene uses a tri-cistronic mechanism to produce three viral proteins, allowing the virus to encode at least 12 proteins in total eight structural and four nonstructural [10]. Research has demonstrated that the σA protein, located within the viral core, exhibits potent anti-interferon (IFN) activity. This protein can bind to dsRNA in a sequence-independent manner, thereby, neutralizing its function and preventing the activation of dsRNA-dependent protein kinase R, which ultimately compromises the antiviral effects of the host IFN system [11]. This immune evasion mechanism has been experimentally validated: in vitro studies have shown that the antiviral state induced by recombinant chicken IFN-α/β in chicken embryo fibroblasts fails to effectively suppress ARV replication [12]. Nevertheless, while certain aspects of ARV's immune escape mechanisms are understood, the corresponding mechanisms in DRV remain largely unknown. In particular, the pathogenicity and immune escape capabilities of the newly emerging NDRV warrant further investigation.

As a core effector molecule of host antiviral innate immunity, type I IFN (IFN-I) plays a pivotal role in establishing early antiviral defense by modulating the expression of IFN-stimulated genes (ISGs) via the JAK-STAT signaling pathway [13, 14]. Specifically, IFN-I first binds to its cell surface receptor (IFNAR), initiating the JAK1/Tyk2 phosphorylation cascade. This process leads to the phosphorylation of STAT1 (Y701) and STAT2 (Y690), enabling their heterodimerization [15]. The resulting complex associates with IFN regulatory factor 9 (IRF9) to form the ISG factor 3 (ISGF3) trimeric complex, which translocates into the nucleus [16]. There, it specifically recognizes the core sequence of the IFN-stimulated response element (ISRE), thereby, inducing robust expression of downstream ISGs [14]. Notably, numerous viruses have evolved sophisticated strategies to antagonize this pathway. For instance, bluetongue virus (BTV) NS3 and NS4 proteins block STAT1 phosphorylation through their SH2 domains [17]. African horse sickness virus (AHSV) NS4 protein disrupts JAK-STAT signaling by interfering with STAT1 phosphorylation and nuclear translocation [18]. Additionally, the μ2 protein of T1L reovirus, which induces viral myocarditis, inhibits IFN signaling by promoting nuclear accumulation of IRF9 [19]. These findings underscore that viral proteins commonly target key nodes in the JAK-STAT signaling pathway as a mechanism for immune evasion. However, the molecular mechanisms underlying NDRV antagonism to IFN-I responses remain incompletely understood and warrant further investigation.

Although the immune evasion mechanism of NDRV directly influences its pathogenicity and the efficacy of vaccine-induced protection, the underlying molecular basis remains poorly understood. In this study, we systematically analyzed the molecular mechanisms by which NDRV antagonizes the IFN-I response and identified a novel immune evasion strategy wherein the σA protein specifically targets key components of the JAK-STAT signaling pathway. Our findings provide new insights into the complex interactions between ARV and the host immune system.

## 2. Materials and Methods

### 2.1. Viruses, Cell Lines, and Antibodies (Abs)

The NDRV strain utilized in this study was isolated from a deceased duckling in Shandong Province, China. LMH and HEK293T cells were cultured in DMEM/F12 basal medium (Procell, PM150312, China) supplemented with 10% FBS (ExCell Biotech, FSP500, China), 100 U/mL penicillin, and 100 μg/mL streptomycin (Sangon Biotech, E607011, China). All cells were maintained under standard culture conditions at 37°C with 5% CO_2_. Polyclonal Abs against NDRV σA were prepared in our laboratory by immunizing rabbits with the corresponding recombinant proteins. Abs against STAT1, STAT2, and β-tubulin were purchased from Proteintech (Catalog Numbers 10144, 16674, and 10094, USA). Abs against IFNAR1 and hemagglutinin (HA) were purchased from ABclonal Technology (Catalog Numbers A18594 and AE105, China). Abs against JAK1, IRF9, p-STAT1, and p-STAT2 were purchased from Cell Signaling Technology (Catalog Numbers 3344T, 28492T, 9167, and 88410T, China).

### 2.2. Plasmid Constructs

The gene encoding the σA protein was amplified from the NDRV genome (GenBank accession no. ON009433) and subsequently cloned into the pCAGGS vector harboring an HA tag, employing standard molecular biology techniques, to generate the HA-σA overexpression plasmid. The constructed vector was sequenced to ensure the absence of any unintended mutations.

### 2.3. RNA Extraction and Quantitative PCR (qPCR)

Total RNA was extracted using TRIzon Total RNA Extraction Reagent (Cwbio, CW0580S, China), and cDNA synthesis was performed using a reverse transcription kit (Cwbio, CW2569M, China). The qPCR reactions were carried out on the LightCycler 96 system (Roche, LC96, USA) with gene-specific primers designed using Primer 5 software. The sequences of the primers and probe used for qPCR are shown in Table 1. Relative gene expression levels were determined using the 2^−ΔΔCT^ method, with GAPDH serving as the reference gene.

### 2.4. Indirect Immunofluorescence Assay

Twenty-Four h postinfection, cells were fixed with 4% paraformaldehyde (Beyotime, P0099, China) for 15 min, followed by permeabilization with 0.2% Triton X-100 (Beyotime, P0096, China) for 10 min. Subsequently, the cells were incubated with 5% bovine serum albumin (BSA) (Beyotime, ST2249, China) for 1 h to block nonspecific binding. All procedures were carried out at room temperature (RT). The cells were then incubated overnight at 4°C with a 1:200 dilution of rabbit anti-NDRV σA polyclonal Ab. After washing, the cells were incubated with FITC-conjugated goat anti-rabbit IgG (1:300 dilution; Transgen, HS111, Germany) for 1 h at RT. Finally, nuclei were stained with Hoechst 33,342 (Beyotime, C1026, China) for 5 min at RT, and the slides were mounted and examined under a fluorescence microscope.

### 2.5. Western Blot Analysis

The recombinant plasmid was transfected into HEK293T cells using Lipo8000TM transfection reagent (Beyotime, C0053, China) following the manufacturer's instructions. Total cellular proteins were extracted using a nuclear and cytoplasmic protein extraction kit (Beyotime, P0027, China), with subsequent separation of cytoplasmic and nuclear fractions. They were lysed in IP lysis buffer (Biosharp, BL509, China) supplemented with protease inhibitors and phosphatase inhibitors for 30 min on ice. Following centrifugation, the supernatant was collected, quantified using the BCA method, and mixed with SDS-PAGE sample loading buffer before being heated at 95°C for 15 min. Electrophoresis was performed at 120 V for 30 min followed by 150 V for 30 min on a 10% SDS-PAGE gel. Proteins were transferred onto PVDF membranes (pore size, 0.22 μm) at 180 mA for 1.5 h. The membranes were blocked with 5% skim milk or 5% BSA in TBST for 1 h at RT and then incubated with primary Abs overnight at 4°C. After three washes with TBST, the membranes were incubated with secondary Ab (1:6000 dilution) for 1 h at RT. Following three additional washes, protein bands were visualized using the ECL chemiluminescence substrate kit (Biosharp, BL523B, China).

### 2.6. Coimmunoprecipitation and Immunoblot Analysis

HEK293T cells were transfected with either an HA-σA expression plasmid or an empty vector. Twenty-Four h post-transfection, the cells were treated with 500 U/mL IFN-α (Beyotime, P5646, China) for 30 min. Following treatment, transfected cells were lysed on ice for 30 min and then boiled in SDS–PAGE sample loading buffer. Cleavage products were separated using 8%–15% SDS–PAGE and subsequently transferred onto a PVDF membrane. The membrane was blocked with 5% skim milk powder and incubated overnight at 4°C with the appropriate dilution of primary Ab. Afterward, the membrane was washed three times with 0.1% PBST (1 × PBS containing 0.1% Tween-20), followed by incubation with a secondary Ab diluted at a ratio of 1:6000 for 1 h at RT. Protein bands were visualized using an ECL chemiluminescence substrate kit (Biosharp, BL523B, China).

For co-IP, 200 μg of cell lysate was incubated with 0.5 μg of mouse anti-HA-Tag Ab at 4°C overnight. Subsequently, a protein A + G agarose gel mixture (Beyotime, P2055, China) was added, and the solution was further incubated for 4 h. After washing the agarose beads, IP lysis buffer was added, and the samples were boiled for 15 min prior to being subjected to western blot analysis.

### 2.7. Luciferase Reporter Assay

HEK293T cells were seeded in 24-well culture plates and transfected with the ISRE luciferase reporter plasmid (pISRE-TA-luc; Beyotime, D2179, China), a control plasmid (pRL-SV40; Beyotime, D2762, China), and either an empty vector or an HA-σA expression plasmid using Lipo8000TM transfection reagent (Beyotime, C0533, China). Twenty-Four h post-transfection, the cells were treated with IFN-α (500 U/mL) for 6 h or remained untreated. Cell lysates were then prepared and assayed for firefly and renilla luciferase activities using a dual luciferase reporter assay kit (Beyotime, RG027, China), following the manufacturer's instructions. Firefly luciferase activity was normalized relative to renilla luciferase activity. All experiments were independently repeated at least three times.

### 2.8. Immunofluorescent Confocal Microscopy

HEK293T cells were seeded in 12-well cell culture plates and transfected with HA-σA or an empty vector for 24 h. Subsequently, the cells were either treated with 500 U/mL of the respective reagent or left untreated for 6 h. Following treatment, the cells were fixed with 4% paraformaldehyde for 15 min at RT. After three washes with PBS buffer, the cell membranes were permeabilized using 0.1% Triton X-100 solution for 20 min. Immunofluorescence staining was performed using a double-label three-color multiplex fluorescent staining kit (Abclonal, RK05902, China), according to the manufacturer's instructions. Fluorescent images were captured using a laser scanning confocal microscope (Olympus, SpinSR, Japan) equipped with a 100x oil immersion objective.

### 2.9. Statistical Analysis

Inter-group statistical comparisons were performed using GraphPad Prism software. Data are expressed as mean ± standard deviation, unless otherwise indicated. Statistical significance is denoted as follows: *⁣*^*∗*^*p* < 0.05, *⁣*^*∗∗*^*p* < 0.01, *⁣*^*∗∗∗*^*p* < 0.001. A *p*-value of less than 0.05 was considered statistically significant.

## 3. Result

### 3.1. Establishment of NDRV Infection Model in HEK293T Cells

**Figure 1:**
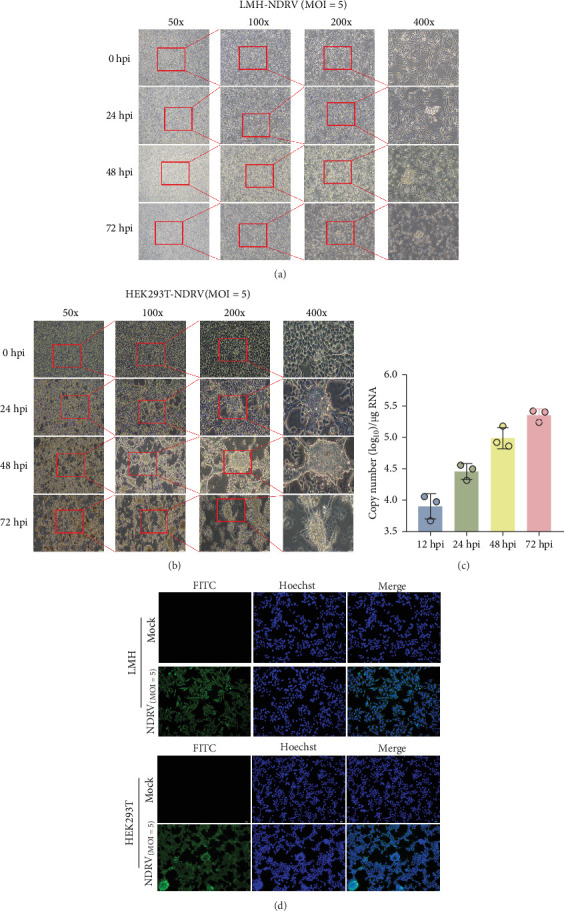
Construction of an HEK293T cell model for NDRV infection. (A) LMH cells were infected with NDRV at MOI = 5 for 24, 48, and 72 h, and cytopathic effects were observed. (B) HEK293T cells were infected under the same conditions, and cytopathic effects were also observed. (C) HEK293T cells were infected for 12–72 h, and total RNA was extracted for qPCR analysis. All tests were repeated at least three times. (D) LMH and HEK293T cells were infected at MOI = 5, and viral protein expression was detected 24 h postinfection using an anti-σA polyclonal antibody.

ARV exhibits the ability to proliferate in a variety of cell lines, including but not limited to DF-1, LMH, HD11, Vero, and BHK-21 [20, 21]. To investigate the interaction mechanism between NDRV and the host innate immune system, we utilized HEK293T cells to establish an in vitro NDRV infection model. Infection and replication kinetics were assessed using IFA and qPCR, respectively. The results showed that after NDRV infection at an MOI of 5, both LMH and HEK293T cells displayed cytopathic effects, including cell shrinkage and exfoliation, by 24 h postinfection (Figure 1A,B). The qPCR data indicated that the mRNA expression level of the NDRV σA protein in HEK293T cells increased significantly over time (Figure 1C). Furthermore, IFA revealed significant fluorescent signals in HEK293T cells after NDRV infection (Figure 1D), suggesting efficient proliferation of NDRV in these cells. Based on these findings, HEK293T cells were selected for subsequent experiments.

### 3.2. NDRV Exhibits Antagonistic Effect on IFN-I-Mediated Antiviral Effects

**Figure 2:**
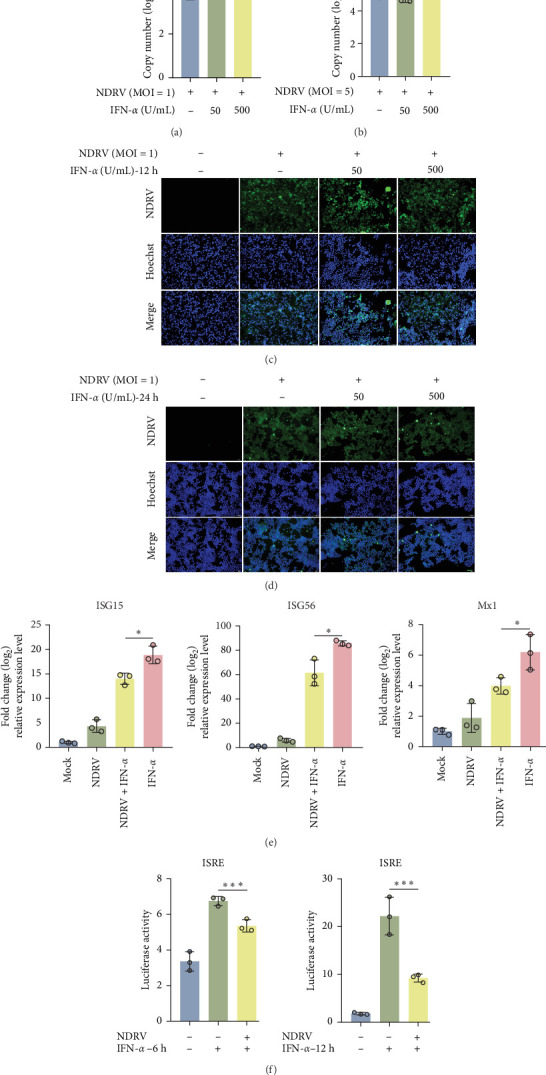
NDRV antagonizes IFN-I mediated antiviral effects. (A, B) HEK293T cells were infected with NDRV (MOI = 1, 5). After 24 h, cells were treated with varying concentrations of IFN-α or left untreated. Viral proliferation was measured by qPCR. All experiments were repeated at least three times. (C, D) HEK293T cells were infected with NDRV at MOI = 1. After 24 h, cells were treated with different concentrations of IFN-α or left untreated. Viral protein expression was analyzed using an anti-σA polyclonal antibody. (E) HEK293T cells were infected with NDRV. After 24 h, cells were treated with varying concentrations of IFN-α or left untreated. A blank group and an IFN-α-treated group served as controls. ISG expression was measured by qPCR. All experiments were repeated at least three times. (F) After NDRV infection of HEK293T cells, pISRE-TA-luc, and pRL-SV40 were cotransfected using Lipo8000TM for 24 h. Cells were either treated with 500 U/mL IFN-α for 6 h or left untreated. Luciferase activity was then measured across at least three independent experiments. Statistical significance is denoted as follows: *⁣*^*∗*^*p* < 0.05, *⁣*^*∗∗*^*p* < 0.01, and *⁣*^*∗∗∗*^*p* < 0.001.

ARV exhibits a significant IFN-antagonistic effect [22]. To evaluate whether NDRV can antagonize the IFN-mediated antiviral immune response, HEK293T cells infected with NDRV were treated with different concentrations of IFN-α (50/500 U/mL) to observe the impact of IFN-α on NDRV proliferation. The results demonstrated that treatment with varying concentrations of IFN-α (50/500 U/mL) did not significantly inhibit the replication level or viral protein expression of NDRV at MOI 1 and MOI 5 (Figure 2A–D). This indicates that NDRV is resistant to the direct antiviral effects of IFN-I.

ISGs are the primary executors of the antiviral effects of IFN-I, and ISRE plays a critical role in regulating ISG expression [14]. Further analysis revealed that while IFN-α treatment markedly upregulated the expression of ISGs (ISG15/56, Mx1), this induction was effectively suppressed by NDRV infection (Figure 2E). Additionally, the ISRE luciferase reporter assay confirmed that NDRV significantly blocked IFN-α-induced ISRE promoter activation (Figure 2F). These findings suggest that NDRV evades the host innate immune response by antagonizing key nodes in the IFN-I signaling pathway.

### 3.3. NDRV σA is an Inhibitor of IFN-I Signaling

**Figure 3:**
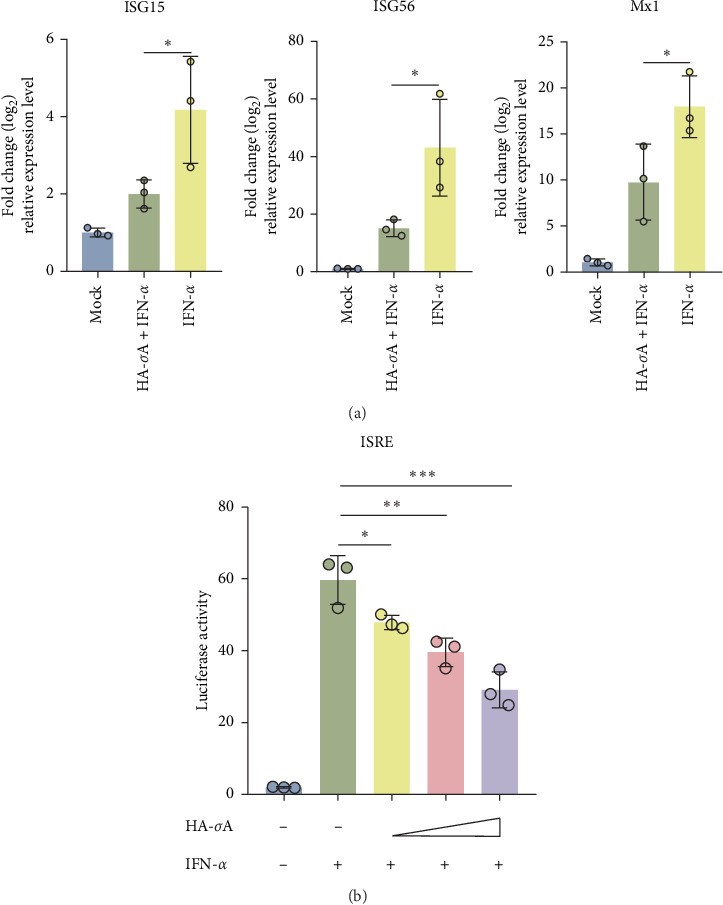
σA is an effector protein of NDRV that antagonizes IFN-I signaling. (A) HEK293T cells were transfected with HA-σA overexpression plasmids or empty vectors for 24 h, then treated with 500 U/mL IFN-α for 12 h. ISG expression was analyzed by qPCR. The experiment was repeated at least three times. (B) HEK293T cells were cotransfected with pISRE-TA-luc, pRL-SV40, and either an empty vector or HA-σA expression plasmid using Lipo8000TM transfection reagent. After 24 h, cells were treated with 500 U/mL IFN-α for 6 h or left untreated. Luciferase activity was then measured in at least three independent assays. Statistical significance is denoted as follows: *⁣*^*∗*^*p* < 0.05, *⁣*^*∗∗*^*p* < 0.01, and *⁣*^*∗∗∗*^*p* < 0.001.

To evaluate the antagonistic effect of the NDRV σA protein on IFN-I, an HA-fuzed σA expression vector was constructed. HEK293T cells were transfected with either the HA-σA construct or an empty vector control for 24 h, followed by treatment with 500 U/mL IFN-α for 12 h. RT-qPCR results demonstrated that IFN-α significantly increased the expression levels of ISG15, ISG56, and Mx1 in the empty vector-transfected group. In contrast, σA significantly suppressed the IFN-α-induced upregulation of these ISGs (Figure 3A). Additionally, HEK293T cells were cotransfected with varying doses of the HA-σA expression vector, the pISRE-TA-luc reporter plasmid, and the pRL-SV40 internal control plasmid. Following 6 h of IFN-α stimulation, luciferase assays revealed that HA-σA transfection significantly inhibited ISRE promoter activation in a dose-dependent manner compared to the empty vector control group (Figure 3B). Collectively, these findings indicate that the σA protein serves as a key effector that enables NDRV to evade the IFN-I-mediated antiviral response.

### 3.4. σA Neither Altering JAK-STAT Protein Expression/Phosphorylation Levels nor Directly Interacting With STAT1/STAT2

**Figure 4:**
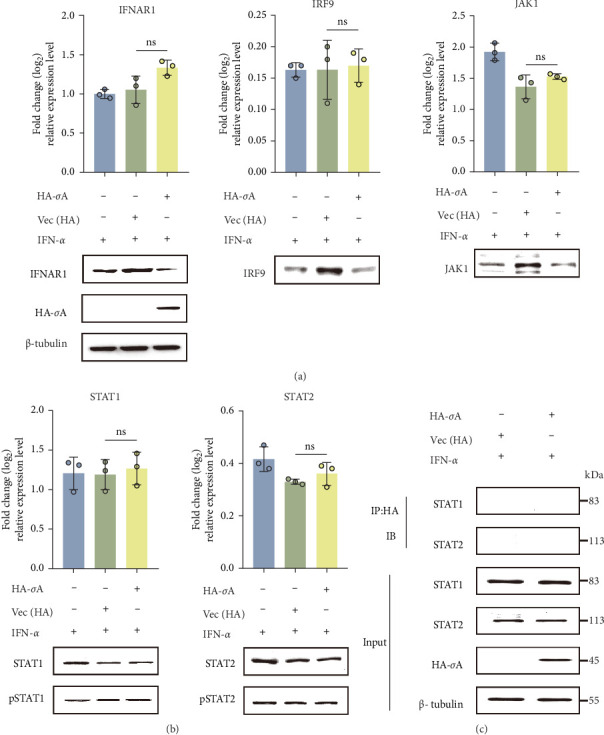
σA does not affect the expression or phosphorylation of proteins related to JAK-STAT signaling pathway, nor does it interact directly with STAT1/STAT2. (A) HEK293T cells were transfected with HA-σA or an empty vector. After 24 h, cells were treated with 500 U/mL IFN-α. Total RNA and protein were extracted, and the expression levels of IFNAR1, IRF9, and JAK1 at the transcriptional and protein levels were analyzed by qPCR and western blot, respectively. (B) Under the same conditions, the transcriptional activity, expression, and phosphorylation of STAT1 and STAT2 were assessed by qPCR and western blot. (C) HEK293T cells were transfected with HA-σA or an empty vector. After 24 h, cells were treated with 500 U/mL IFN-α. Total protein lysates were immunoprecipitated with anti-HA antibody and analyzed by western blot using anti-STAT antibodies.

Viral proteins typically achieve immune escape by targeting key components of the JAK-STAT signaling pathway and antagonizing IFN-I signaling [23, 24]. To investigate the impact of the NDRV σA protein on the JAK-STAT signaling pathway, HA-σA was transfected into HEK293T cells, followed by stimulation with 500 U/mL IFN-α 24 h later. The mRNA levels of key pathway components were analyzed using RT-qPCR, while the expression and phosphorylation status of relevant proteins were assessed by western blot. The results demonstrated that HA-σA did not significantly alter the transcription or expression levels of critical proteins in the JAK-STAT pathway—such as IFNAR1, STAT1, STAT2, and IRF9—compared to the empty vector control (Figure 4A,B). Furthermore, no significant differences were observed in IFN-α-induced phosphorylation of STAT1 (pSTAT1) or STAT2 (pSTAT2) between the experimental and control groups (Figure 4B). These findings indicate that NDRV σA does not interfere with the initial activation of the JAK-STAT signaling pathway.

Given that many viral proteins interfere with the function of STAT molecules by directly binding to them [25], we further analyzed the potential interaction between NDRV σA and STAT proteins using a co-IP assay. HA-σA was transfected into HEK293T cells, and 24 h later, the cells were stimulated with 500 U/mL IFN-α. Total protein lysates were immunoprecipitated with an anti-HA Ab and subsequently analyzed by western blotting using specific Abs against STAT proteins. The results demonstrated that HA-σA did not form complexes with either STAT1 or STAT2 (Figure 4C). These findings suggest that σA regulates signaling through a mechanism that does not involve direct binding to or degradation of STAT proteins.

### 3.5. σA Specifically Blocks Nuclear Translocation of STAT1/STAT2

**Figure 5:**
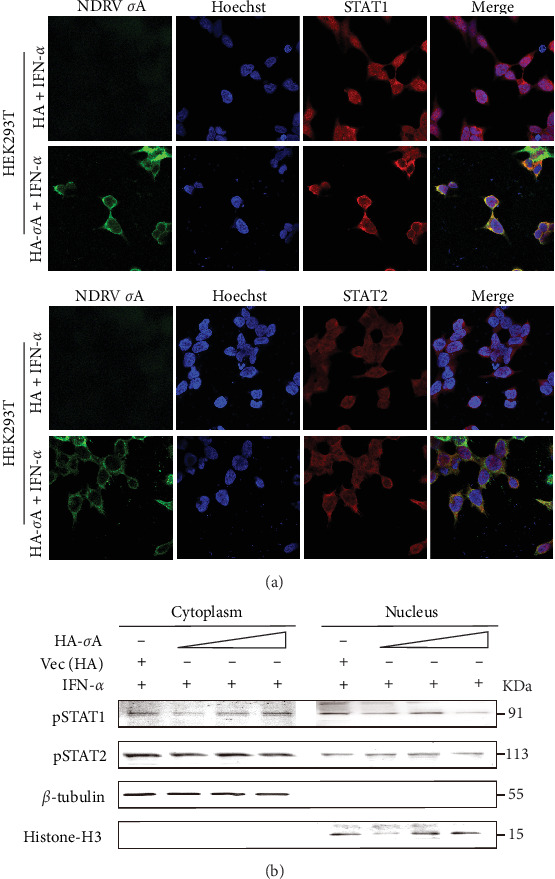
σA protein inhibits IFN-α-induced STAT1/STAT2 nuclear translocation. (A) HEK293T cells were transfected with HA-σA plasmid or empty vector. After 24 h, cells were treated with 500 U/mL IFN-α, and the subcellular localization of STAT1/STAT2 was analyzed by confocal microscopy. (B) HEK293T cells were transfected with HA-σA plasmid or empty vector. After 24 h, cells were treated with 500 U/mL IFN-α. Nuclear and cytoplasmic fractions were isolated, and STAT1/STAT2 phosphorylation was assessed by western blot.

To clarify the effect of σA protein on downstream events in the JAK-STAT signaling pathway, we systematically evaluated its regulatory impact on STAT1/STAT2 nuclear translocation using IFA and western blot. HA-σA plasmid and its corresponding empty vector were transfected into HEK293T cells, followed by treatment with 500 U/mL IFN-α 24 h post-transfection. The subcellular localization of STAT1/STAT2 was then examined via laser confocal microscopy. Results demonstrated that IFN-α stimulation significantly induced the nuclear accumulation of both STAT1 and STAT2. However, in cells expressing σA, the nuclear translocation of STAT1/STAT2 was markedly inhibited, resulting in a significant reduction in their nuclear accumulation (Figure 5A).

To further quantify this observation, we conducted a nucleoplasmic fractionation assay combined with western blot to assess the subcellular distribution of phosphorylated STAT proteins. The results revealed that although IFN-α effectively induced phosphorylation of STAT1 and STAT2, the nuclear levels of pSTAT1 and pSTAT2 exhibited a dose-dependent decrease in HA-σA transfected cells (Figure 5B). Collectively, these findings indicate that NDRV σA suppresses IFN-I signaling activation by specifically interfering with the nuclear translocation of STAT1/STAT2, without affecting their phosphorylation status.

## 4. Discussion

**Figure 6:**
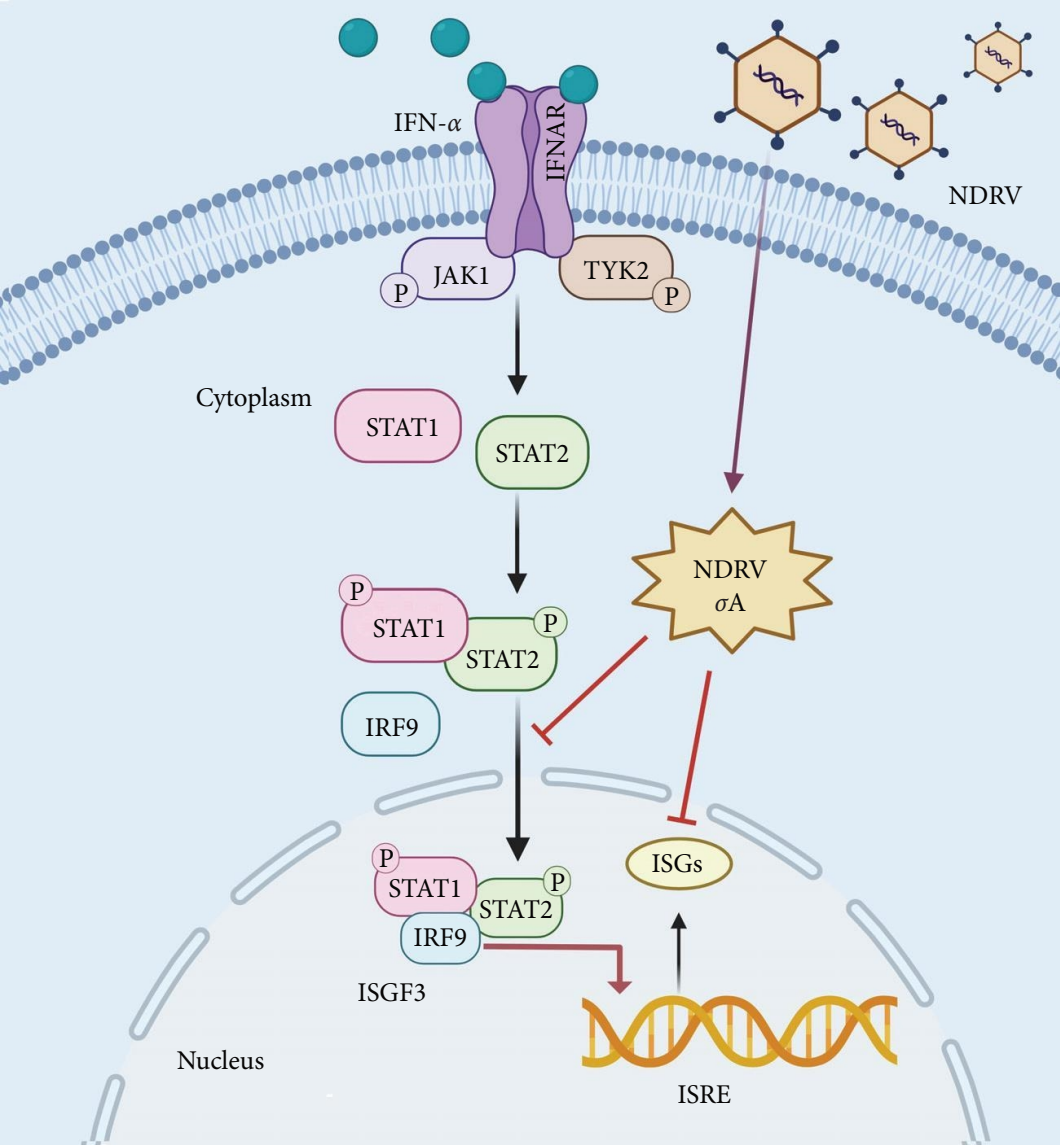
Schematic diagram illustrating the mechanism through which the NDRV σA protein inhibits the nuclear translocation of STAT1/STAT2. The σA protein significantly suppresses the IFN signaling pathway and its associated antiviral effects by specifically blocking the nuclear translocation of STAT1/STAT2, thereby, enabling the virus to evade the host's innate immune response.

As an immunosuppressive pathogen, NDRV infection compromises the host immune system, induces immunosuppression in the host, and interferes with the immune efficacy of other vaccines, thereby, causing substantial economic losses to the duck farming industry [5, 7]. However, the understanding of the mechanisms by which NDRV antagonizes the host's innate immune response remains limited. In this study, we identified a novel immune escape mechanism employed by NDRV, wherein its σA protein specifically inhibits the nuclear translocation of STAT1 and STAT2. Our findings demonstrate that although NDRV infection can partially activate the IFN-I-mediated antiviral immune response, it exerts a significant suppressive effect on this response. The σA protein attenuates virus-induced IFN-I signaling by specifically blocking the nuclear translocation of STAT1 and STAT2 (Figure 6).

The innate immune response serves as the first line of defense against pathogen invasion in the host [26]. Upon infection, pathogen-associated molecular patterns (PAMPs) are recognized by pattern recognition receptors (PRRs), which subsequently activate the NF-κB signaling pathway and promote the transcription of IRFs [27]. These IRFs translocate into the nucleus and induce the expression of IFN-I. The IFN-I produced by pathogen stimulation does not have direct antiviral activity. Instead, it must bind to IFNAR, activate the JAK-STAT signaling pathway, transmit the signal into the cell, and upregulate various ISGs to exert antiviral effects [28]. Numerous studies have demonstrated that ARV is capable of inhibiting the IFN-mediated antiviral response [29]. Specifically, ARV-encoded proteins μB, σA, σB, and σC all exhibit significant inhibitory effects on IFN-I induction, with σA being the key viral protein responsible for this antagonistic activity [30]. Our study confirms that despite the notable sequence divergence in the σA gene between NDRV and ARV [5], the σA protein of NDRV also possesses a strong capacity to inhibit IFN-I signaling. It significantly suppresses IFN-I-induced ISRE promoter activation and the upregulation of ISG expression levels. Previous research has revealed that the σA protein of ARV antagonizes IFN-I production through multiple mechanisms [29, 31]. It potently inhibits TLR3- and MDA5-mediated activation of IFN-β and suppresses IFN-β promoter activation induced by Poly(I:C) and Sendai virus (SeV) in a dose-dependent manner [32]. Moreover, σA interacts with IRF7, thereby, preventing its dimerization and nuclear translocation, ultimately suppressing IFN-β production [32]. The antagonistic effect of σA on IFN-I is closely associated with its dsRNA binding ability. By nonsequence-specifically binding to dsRNA, σA neutralizes its stimulatory effect, thereby, blocking PKR activation [30]. Furthermore, arginine residues 155 and 273 were identified as essential for both the anti-IFN activity and nuclear localization of the σA protein. Mutation of the dsRNA binding site in σA resulted in reduced electrophoretic mobility of PKR and its phosphorylation. Further investigations are required to elucidate the functional domains of the NDRV σA protein.

In ARV and DRV, the σA protein is encoded by the S2 gene, whereas in Muscovy DRV (MDRV), the σA protein is encoded by the S1 gene. However, the full-length sequence and the size of the coding DNA sequence (CDS) region are identical across all three viruses [33]. The MDRV σA protein can up-regulate the expression of IFN-I and related signaling pathway factors induced by MDRV infection. Arginine residues 155 and 273 of the σA protein have been identified as key regulatory sites involved in IFN-I modulation. Nevertheless, transfection with σA protein alone did not significantly induce IFN-I expression. Similarly to the ARV σA protein, the μ1 protein of mammalian reovirus (MRV) exerts a significant inhibitory effect on MRV-induced IFN-β production. The μ1 protein suppresses RLR-mediated signal transduction by reducing the expression levels of RIG-I, MDA5, MAVS, and TBK1, and further inhibits IFN-β production by blocking the phosphorylation and nuclear translocation of IRF3 [34]. In addition, MRV can interfere with signal transduction by suppressing NF-κB activation. MRV infection reduces the expression of the upstream activator IKKβ and the NF-κB essential modulator, leading to down-regulation of NF-κB expression and subsequent inhibition of IFN production [35].

The JAK-STAT signaling pathway acts as the central conduit for initiating antiviral immune responses upon IFN activation. Viruses can antagonize IFN-I-mediated JAK-STAT signal transduction through multiple mechanisms, primarily by employing viral proteins and genomic elements that specifically target key regulatory components of the JAK-STAT pathway. For instance, the NS3 protein of BTV inhibits IFN signaling by preventing STAT1 phosphorylation and promoting the autophagic degradation of STAT2 [36]. Similarly, the μ2 protein of T1L reovirus contributes to viral myocarditis by suppressing IFN signaling through the induction of nuclear accumulation of IRF9 [19]. Herpesvirus-encoded LMP2A and LMP2B proteins interfere with IFN signaling by targeting IFNAR for accelerated degradation [37]. In contrast to these previously described inhibitory mechanisms, the NDRV σA protein does not influence the expression or phosphorylation status of transcription factors involved in the JAK-STAT pathway, nor does it interact directly with STAT1 or STAT2. Instead, NDRV σA exerts a unique mechanism of immune evasion by impairing the nuclear translocation of STAT1/STAT2 following IFN-α stimulation. This is achieved through the inhibition of STAT1 and STAT2 phosphorylation, resulting in reduced nuclear accumulation of ISGF3 and subsequent suppression of IFN-I signal transduction. Similar mechanisms are observed with the NS4 protein of AHSV, which disrupts the JAK-STAT pathway by interfering with both the phosphorylation and nuclear translocation of STAT1 [18]. Additionally, BTV suppresses IFN signaling by inhibiting STAT1 phosphorylation and nuclear entry [17].

In addition to viral proteins directly regulating the JAK-STAT signaling pathway, recent studies have revealed that host microRNAs also play a crucial role in viral immune escape [38]. Notably, host microRNAs exhibit bidirectional regulation during NDRV infection. On one hand, NDRV infection significantly induces the expression of miR-155-5p in DEF cells. Research indicates that miR-155-5p targets the 3′-UTR of suppressor of cytokine signaling 5 (SOCS5), thereby, suppressing its expression. This leads to reduced IRF7 expression, decreased IFN production, and consequently enhanced NDRV replication [39]. On the other hand, certain host miRNAs exert antiviral effects. For instance, ARV infection markedly upregulates gga-miR-30c-5p expression in DF-1 cells. The elevated levels of gga-miR-30c-5p inhibit autophagy induced by ARV through targeting autophagy-related protein 5 (ATG5), as well as suppress virus-induced syncytial formation, ultimately limiting viral replication [40]. These findings not only deepen our understanding of the complexity of virus-host interactions but also offer novel insights for the development of microRNA-based antiviral therapeutic strategies.

In conclusion, compared to avian influenza virus, Newcastle disease virus, and other pathogens, the mechanisms through which ARV (especially NDRV) evades host immune clearance and antagonizes host immune responses remain insufficiently understood and warrant further investigation. Future studies focusing on NDRV's interference with TLR-mediated signal transduction, suppression of IFN-I signaling pathways, regulation of inflammatory responses, and induction of host cell damage will contribute to a scientific basis for disease prevention and control strategies, as well as the development of novel vaccines. These efforts are expected to aid in mitigating, and potentially eliminating, the impact of ARV on the global poultry industry.

## Figures and Tables

**Table 1:** Primers used in this study.

Primer name	Sequence (5′–3′)	
	Forward	Reverse
NDRV-S2	CCCGGATTCTCGATGAATGGT	CGACCCACTGCTGGATACAAG
NDRV-S2-probe	FAM-5′r-AACGCCTGTGCACGAGCTGAAC-3′-TAMRA r	
HA-σA	CCAGATTACGCTGAATTCATGGCGCGTGCCGTGTAC	AAGATCTGCTAGCTCGAGGGCGGTAAAAGTGGCTAG
ISG15	GGGACCTGACGGTGAAGATG	CGCCGATCTTCTGGGTGAT
ISG56	GCTTTCAAATCCCTTCCGCTAT	GCCTGGCCCGTTCATAAT
Mx1	CCATCGGAATCTTGACG	GCTTCGGACAGGCTCAG
GAPDH	AAAATCAAGTGGGGCGATGCT	GGGCAGAGATGATGACCCTTT
IFNAR1	ATTTACACCATTTCGCAAAGCTC	TCCAAAGCCCACATAACACTATC
IRF9	GCCACCAGGAATCGTCTCTG	AGGGACTGAGTGTGCAGTTC
JAK1	CCACTACCGGATGAGGTTCTA	GGGTCTCGAATAGGAGCCAG
STAT1	CAGCTTGACTCAAAATTCCTGGA	TGAAGATTACGCTTGCTTTTCCT
STAT2	CTGCTAGGCCGATTAACTACCC	TCTGATGCAGGCTTTTTGCTG

*Note:* The underlined nucleotide sequences in the primer sequence represent the homologous arm sequences of the vector.

## Data Availability

The data are available upon request from the authors, Hongzhi Wang (whz20220904@swu.edu.cn), upon reasonable request.
